# Dietary exposure levels to ^134^Cs, ^137^Cs, ^90^Sr, and ^239+240^Pu in Japan after the Fukushima Daiichi Nuclear Power Plant accident: a duplicate portion study for fiscal years 2012–2014

**DOI:** 10.1265/ehpm.25-00072

**Published:** 2025-06-25

**Authors:** Hiroshi Terada, Ikuyo Iijima, Sadaaki Miyake, Tomoko Ota, Ichiro Yamaguchi, Hiroko Kodama, Hideo Sugiyama

**Affiliations:** 1 National Institute of Public Health, 2-3-6 Minami, Wako-shi, Saitama 351-0197, Japan; 2 Kanagawa Prefectural Institute of Public Health, 1-3-1 Shimomachiya, Chigasaki-shi, Kanagawa 253-0087, Japan; 3 Saitama Prefectural Institute of Public Health, 410-1 Ewai, Yoshimi-machi, Hiki-gun, Saitama 355-0133, Japan; 4 Japan Chemical Analysis Center, 295-3 Sanno-cho, Inage-ku, Chiba-shi, Chiba 263-0002, Japan; 5 Teikyo Heisei University, 2-51-4 Higashiikebukuro, Toshima-ku, Tokyo 170-8445, Japan

**Keywords:** Duplicate portion study, Dietary intake, Committed effective dose, Cesium-134, Cesium-137, Strontium-90, Plutonium, Standard limit, Fukushima accident

## Abstract

**Background:**

Since the accident at Fukushima Daiichi Nuclear Power Plant (FDNPP), concerns have arisen in Japan regarding the presence of radionuclides in food. Moreover, exposure levels to ^90^Sr and Pu isotopes in adults and those to ^134^Cs+^137^Cs, ^90^Sr, and Pu (where Cs, Sr, and Pu are cesium, strontium, and plutonium, respectively) in children have not been examined. Therefore, this study employed a duplicate portion approach to examine dietary exposure levels of radionuclides in adults and children following the FDNPP accident.

**Methods:**

The study spanned fiscal years 2012–2014 and was conducted in 10 prefectures: Hokkaido, Iwate, Miyagi, Fukushima, Ibaraki, Saitama, Tokyo, Kanagawa, Osaka, and Kochi. The participants provided portions of their meals for two non-consecutive days and completed questionnaires on the meal items. The activity concentrations of ^134^Cs, ^137^Cs, ^90^Sr, and ^239+240^Pu, which are targets of standard limits for radionuclides in foods in Japan, were determined according to the Radioactivity Measurement Series. The daily intake was calculated based on the radionuclide activity concentrations in the duplicate portion samples, and the committed effective doses were estimated using dose coefficients for the ingestion of each radionuclide provided by the International Commission on Radiological Protection.

**Results:**

Approximately 80 duplicate samples were obtained in each fiscal year, and 242 samples were collected. The highest summed activity concentration of ^134^Cs and ^137^Cs was 11 Bq/kg, which was recorded in Date City (child) in 2013; this level was approximately one-ninth of the standard limit for general foods (100 Bq/kg). The committed effective dose from annual ingestion of the sample described above was 74 µSv, approximately 14 times lower than the maximum permissible level of 1 mSv/y. Pu was not detected and the ^90^Sr activity concentrations were similar to those before the FDNPP accident.

**Conclusions:**

For the samples examined in the present study, the ^134^Cs, ^137^Cs, ^90^Sr, and ^239+240^Pu dietary exposure levels were considerably lower than the regulatory levels and may not pose a health risk.

**Supplementary information:**

The online version contains supplementary material available at https://doi.org/10.1265/ehpm.25-00072.

## Background

Fourteen years have passed since the occurrence of the Great East Japan Earthquake on March 11, 2011, and more than three years have passed since Russia invaded Ukraine on February 24, 2022. The Mw 9.0 earthquake and the subsequent tsunami caused a nuclear accident at the Fukushima Daiichi Nuclear Power Plant (FDNPP) of the Tokyo Electric Power Company. The current situation in Ukraine poses a risk of another nuclear disaster.

The FDNPP accident was the most severe nuclear disaster since the 1986 Chornobyl NPP disaster. The Japanese Nuclear and Industrial Safety Agency has rated the FDNPP accident as Level 7 (the most serious) on the International Nuclear and Radiological Event Scale [1]; this implies considerable radionuclide release with widespread adverse health and environmental effects.

Humans can be exposed to radionuclides through food consumption, and can experience adverse health effects due to the emitted ionizing radiation. In the early stages of a nuclear disaster, the presence of radioactive iodine isotopes, such as iodine-131 (^131^I), is the most critical issue, as those isotopes potentially increase the risk of thyroid cancer among children [2, 3]. The presence of cesium-134 (^134^Cs), cesium-137 (^137^Cs), strontium-90 (^90^Sr), and plutonium (^238^Pu, ^239^Pu, ^240^Pu, and ^241^Pu) isotopes is a long-term concern, as they have longer half-lives than ^131^I (Supplementary Table S1).

The Ministry of Health, Labour and Welfare (MHLW) has established standard limits; for example, the total effective dose due to radionuclides should not exceed the maximum permissible dose of 1 mSv/y [4]. Radionuclides with half-lives of >1 year, namely, ^134^Cs, ^137^Cs, ^90^Sr, Pu isotopes, and ruthenium-106 (^106^Ru) (Supplementary Table S1), were selected for regulation as the limits were implemented on April 1, 2012, one year after the FDNPP accident [5, 6]. The limits were set to 100, 50, and 10 Bq/kg for general foods, milk and infant foods, and drinking water, respectively [4]. Milk and infant foods are categorized separately from general foods because the Food Safety Commission of Japan suggests that children may be more susceptible to radiation than adults [5, 6]. Radionuclides in foods are monitored by taking the summed activity concentration of ^134^Cs and ^137^Cs (^134^Cs+^137^Cs) as an indicator; these radionuclides were selected because measurement of other radionuclides is time consuming. Although local governments have conducted numerous inspections since the FDNPP accident, data on other radionuclides are scarce.

In our previous study, the average dietary exposure levels to radionuclides in the adult population in Japan were estimated using total diet studies (TDSs) [7]. The committed effective doses (CEDs) due to ^134^Cs+^137^Cs in fiscal year (FY) 2011 were 12, 17, and 3.8 µSv in Sendai City, Fukushima City, and Tokyo, respectively, all of which were two orders of magnitude higher than those before the FDNPP accident (0.022–0.15 µSv) but well below the maximum permissible dose [7]. However, exposure levels to ^90^Sr and Pu isotopes in adults and those to ^134^Cs+^137^Cs, ^90^Sr, and Pu in children have not been examined.

This study evaluates dietary intake of ^134^Cs+^137^Cs, ^90^Sr, and ^239+240^Pu in children and adults in Japan. We use the duplicate portion (DP) approach instead of TDSs because DP studies are ideal for estimating actual exposure of individuals to contaminants [8, 9]. Most of the data have already been reported in a grant research report [10]. In this paper, we summarize the results and conducted statistical analysis on the differences in radionuclide exposure levels among regions, age groups and FYs. We also reviewed the results of other studies conducted in Japan after the FDNPP accident.

## Methods

### Duplicate-diet sampling and preparation

This study was approved by the Ethics Committee of the National Institute of Public Health (NIPH-IBRA #12042) and conducted in accordance with the Ethical Guidelines for Epidemiological Research of the MHLW and Ministry of Education, Culture, Sports, Science and Technology of Japan. The participants were informed of the study purpose and provided written informed consent.

**Fig. 1:**
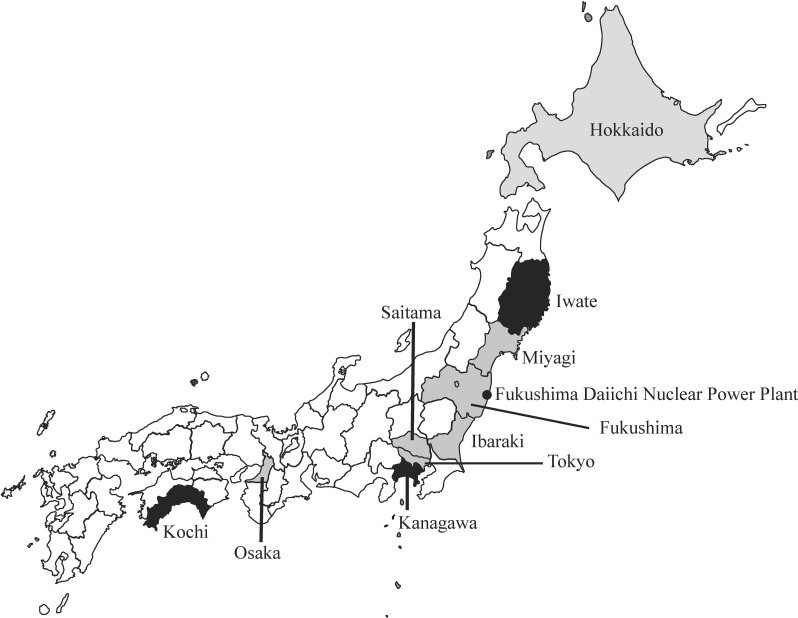
Sampling areas for the duplicate portion study in Japan during fiscal years 2012–2014 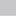
: for adults (20 years and above) and children aged three to six years 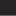
: for adults (20 years and above) This study was conducted in ten prefectures of Japan. Iwate, Miyagi, Fukushima, Ibaraki, Saitama, Tokyo, and Kanagawa were together considered the Tohoku–Kanto region. Hokkaido, Osaka, and Kochi were considered the other regions.

Figure 1 shows the sampling areas considered in this study. DP samples for adults were obtained from participants aged >20 years living in nine prefectures—Hokkaido, Iwate, Miyagi, Ibaraki, Saitama, Tokyo, Kanagawa, Osaka, and Kochi—and six cities in Fukushima Prefecture—Soma, Minamisoma, Date, Fukushima, Koriyama, and Aizuwakamatsu. The seven prefectures near the FDNPP—Iwate, Miyagi, Fukushima, Ibaraki, Saitama, Tokyo, and Kanagawa—were classified as being in the Tohoku–Kanto region. The other three prefectures, which are more than 300 km from the FDNPP, were classified as “other” regions. The child samples were obtained for each FY from households having children aged three to six years in all the locations mentioned above, excluding the Iwate, Kanagawa, and Kochi Prefectures. The participant households provided duplicates of all foods, drinking water, beverages, and snack foods consumed by them or their children on two non-consecutive days; the participants completed questionnaires (Supplementary Tables S2 and S3).

The study participants were recruited via local governments, pediatricians, or preschools. The network of co-authors recruited families in each location. DP samples were provided by the same household for three FYs: FY 2012 (March 2013), FY 2013 (September–November 2013), and FY 2014 (December 2014–March 2015). However, households in which children reached the age of seven years were replaced. Approximately 80 DP samples were obtained for each FY, yielding a total of 242 samples. The total numbers of DP samples from the Tohoku–Kanto region and “other” regions were 197 and 45, respectively, and those for adults and children were 135 and 107, respectively. The questionnaire return rate was 87%.

Individual DP samples were mixed and homogenized using a blender (Model 990; Hamilton Beach Commercial, North Carolina, USA). The homogenates were freeze- or heat-dried, and incinerated at 450 °C for 24 h using an electric muffle furnace (FUW263PA of Advantec Toyo Kaisha Ltd., Tokyo, Japan).

### Analysis of ^137^Cs, ^134^Cs, ^90^Sr, and ^239+240^Pu

The ^137^Cs and ^134^Cs activity concentrations in the ash samples were analyzed in accordance with the “Gamma-ray Spectrometry Using Germanium Detectors” standard of the Nuclear Regulation Authority (NRA) [11]. Each sample was placed in a 100-mL plastic container and the activities were determined using high-purity Ge semiconductor detectors (GC2018 and GC3520, Canberra Co., Connecticut, USA; EGPC 20-190-R, EURYSIS Co., Lingolsheim, Cedex-France; CNVDS30-35195, Oxford Co., Oxford, UK; GEM40-76, ORTEC Co., Tennessee, USA; and IGC40200, Princeton Gamma-Tech Instruments, Inc., New Jersey, USA) connected to a multichannel analyzer (DSA1000, Canberra Co., MCA8000, Princeton Gamma-Tech Instruments, Inc., or MCA 7600, Seiko EG&G Co., Ltd., Tokyo, Japan). A count time exceeding 200,000 s was used for sample measurement to achieve a very low limit of detection (LOD), which was a maximum of 0.05 Bq/kg for ^137^Cs and ^134^Cs. The gamma spectrometry systems were calibrated using γ-ray volume sources (MX033U8PP and MX033MR, Japan Radioisotope Association, Tokyo, Japan). Gamma-ray spectrometry quality assurance was performed using comparison analysis, as described previously [12].

The ^90^Sr activity concentrations were determined according to the “Radiostrontium Analysis” standard [13]. A stable Sr carrier was added to the ash samples, which were then dissolved through heating in aqua regia and nitric acid. After digestion, Sr was precipitated from the solution as an insoluble carbonate. Subsequently, the Sr was purified using a 100–200-mesh cation-exchange resin (Dowex^®^ 50 W-X8, Dow Chemical Co., Michigan, USA). Yttrium-90 (^90^Y), the progeny radionuclide of ^90^Sr, was removed from the solution via hydroxide precipitation. The solutions were stored for two weeks to facilitate radioactive equilibrium between the ^90^Sr and ^90^Y. Then, the ^90^Y was co-precipitated using iron hydroxide. The ^90^Y activity was measured for 3,600 s using a low-background anti-coincidence beta counter (model LBC-471Q, Aloka Co., Ltd., Tokyo, Japan), whereas that of ^90^Sr was calculated based on chemical yields determined using inductively coupled plasma atomic emission spectrometry (model SPS7800, SII Nano Technology Inc., Tokyo, Japan).

The “Plutonium Analysis” standard was used to measure the ^239^Pu and ^240^Pu activity concentrations [14]. Briefly, ash samples were digested with nitric acid and heated following the addition of ^242^Pu as a yield tracer. The Pu was separated and purified using a 100–200-mesh anion-exchange resin column (Dowex^®^ 1-X8 Dow Chemical Co.) and electrodeposited on a stainless-steel disc for measurement. The ^239+240^Pu activity was determined for 80,000 s using silicon semiconductor detectors (BU-020-450-AS; Ortec Co., Tennessee, USA).

The activities of the above-mentioned radionuclides, except ^239+240^Pu, were corrected to the ends of their sampling periods. Those of ^239+240^Pu were not corrected because of their long half-lives (24,110 and 6,561 y for ^239^Pu and ^240^Pu, respectively). The activity concentrations in the DP samples were expressed as “becquerels of radionuclides per kilogram of fresh sample.”

### Dietary intakes and CEDs

The daily dietary intakes of ^137^Cs, ^134^Cs, ^90^Sr, and ^239+240^Pu by individuals (Bq/d/person) were obtained by multiplying the activity concentrations of these radionuclides in each DP sample (Bq/kg) by the daily food consumption per person (kg/d/person), which was calculated from the weight of the DP sample (kg/person) divided by the 2-d sampling period.

The individual CED of exposure from one year of DP-sample consumption was calculated as follows:

$$D_{r}=I_{r}\times C_{ra}\times 365$$

where *D_r_* is the CED due to radionuclide *r* (Sv), *I_r_* is the individual intake of *r* (Bq/d/person), *C_ra_* is the age-specific dose coefficient for *r* and age group *a* via ingestion (Sv/Bq), and 365 is the DP-sample consumption period (d). The International Commission on Radiological Protection specifies the dose coefficients for adults and children (5 years old) as 1.3 × 10^−8^ and 0.96 × 10^−8^ Sv/Bq for ^137^Cs, 1.9 × 10^−8^ and 1.3 × 10^−8^ Sv/Bq for ^134^Cs, 2.8 × 10^−8^ and 4.7 × 10^−8^ Sv/Bq for ^90^Sr, and 25 × 10^−8^ and 33 × 10^−8^ Sv/Bq for ^239+240^Pu, respectively [15].

The activity concentrations, dietary intake, and CEDs were calculated assuming values of half the LODs for non-detects.

### Statistical analyses

We analyzed the differences in radionuclide exposure levels among regions, age groups, and FYs using forward-backward stepwise multiple regression. Log-transformed values were used for intakes and CEDs as they were right-skewed distributions. However, because of the small sample size for ^90^Sr, comparisons between adults and children were made using a Welch t-test. Statistical analyses were performed using R, version 4.3.1 [16].

## Results and discussion

### ^134^Cs and ^137^Cs

Table 1 lists the ^134^Cs, ^137^Cs, and ^134^Cs+^137^Cs activity concentrations in the DP samples. ^134^Cs and ^137^Cs were detected in 135 and 208 of the 242 samples, respectively. The detection rates of ^134^Cs, which has a shorter half-life than that of ^137^Cs (2.1 and 30.1 y, respectively), were lower than those of ^137^Cs, and decreased remarkably from FY 2012 to 2014. Higher ^134^Cs and ^137^Cs activity concentrations were obtained in the Tohoku–Kanto region than in the other regions.

**Table 1:** Activity concentrations of ^134^Cs and ^137^Cs in Japan estimated using the duplicate portion study

**Radionuclide**	**Region^1^**	**Age group^2^**	**FY^3^**	**n**	**Range^4^**	**Mean^5^**	**SD^5^**	**Detection rate (%)**
^134^Cs (Bq/kg)	Tohoku–Kanto	Adults	2012	36	<0.010–0.23	0.049	0.049	78
			2013	36	<0.013–0.40	0.051	0.073	78
			2014	36	<0.011–0.10	0.024	0.024	50

		Children	2012	31	<0.014–0.50	0.071	0.10	68
			2013	29	<0.017–3.4	0.15	0.63	59
			2014	29	<0.011–0.082	0.026	0.020	48

	Others	Adults	2012	9	<0.006–0.056	0.020	0.020	33
			2013	9	<0.017–0.040	0.017	0.011	44
			2014	9	<0.012–0.030	0.012	0.007	11

		Children	2012	6	<0.013–0.016	0.012	0.003	17
			2013	6	<0.020–0.018	0.013	0.003	0
			2014	6	<0.023–0.017	0.015	0.002	0

^137^Cs (Bq/kg)	Tohoku–Kanto	Adults	2012	36	<0.020–0.42	0.088	0.093	78
			2013	36	0.02–0.91	0.12	0.16	100
			2014	36	<0.011–0.37	0.081	0.080	97

		Children	2012	31	<0.021–0.94	0.14	0.19	84
			2013	29	0.023–7.9	0.35	1.4	100
			2014	29	<0.017–0.29	0.089	0.071	90

	Others	Adults	2012	9	<0.024–0.12	0.048	0.042	78
			2013	9	<0.014–0.085	0.041	0.024	78
			2014	9	<0.013–0.085	0.029	0.025	67

		Children	2012	6	<0.013–0.036	0.015	0.011	33
			2013	6	<0.014–0.038	0.021	0.013	50
			2014	6	<0.018–0.035	0.022	0.012	50

^134^Cs + ^137^Cs (Bq/kg)^4^	Tohoku–Kanto	Adults	2012	36	0.018–0.65	0.14	0.14	-
			2013	36	0.028–1.3	0.17	0.23	-
			2014	36	0.013–0.47	0.10	0.10	-

		Children	2012	31	0.019–1.4	0.21	0.29	-
			2013	29	0.036–11	0.50	2.1	-
			2014	29	0.014–0.38	0.11	0.091	-

	Others	Adults	2012	9	0.021–0.17	0.067	0.060	-
			2013	9	0.017–0.13	0.058	0.033	-
			2014	9	0.015–0.12	0.041	0.031	-

		Children	2012	6	0.013–0.049	0.028	0.012	-
			2013	6	0.017–0.056	0.033	0.015	-
			2014	6	0.023–0.048	0.037	0.011	-

^1^The Tohoku–Kanto region includes Iwate, Miyagi, Fukushima, Ibaraki, Saitama, Tokyo, and Kanagawa, and the others include Hokkaido, Osaka, and Kochi.

^2^Adults and children were persons aged 20 years and over, and aged three to six years, respectively.

^3^Fiscal year. The sampling periods for FY 2012, FY 2013, and FY 2014 were March 2013, September–November 2013, and December 2014–March 2015, respectively.

^4^Upper limits were calculated using half the limits of detection for non-detects.

^5^Calculated using half the limits of detection for non-detects.

The highest ^134^Cs+^137^Cs activity concentration (11 Bq/kg) was found in the food sample of a child from the Tohoku–Kanto region (Date City) in FY 2013; this was one-ninth the standard limit for general foods (100 Bq/kg). The questionnaire data showed that the household consumed homegrown foods. Tsubokura et al. [17] reported high ^134^Cs+^137^Cs levels in Fukushima Prefecture residents who consumed homegrown products; their Cs levels decreased remarkably after they were advised to consume distributed food. Distributed foods are tested before entering the market but homegrown products are not. In FY 2013, local-government radioactive monitoring showed that only seven of 28,986 distributed foods exceeded the standard limits [18]. Furthermore, households in Tohoku–Kanto region who consumed homegrown foods exhibited significantly higher dose due to radionuclides than those who did not (p < 0.006) even though excluding exceptionally high one. These findings strongly suggested that the high concentration in the DP sample mentioned above was due to homegrown foods.

The ^134^Cs+^137^Cs dietary exposure results are summarized in Table 2. Significantly higher ^134^Cs+^137^Cs intake was recorded in the Tohoku–Kanto region compared to the other regions (p < 7 × 10^−10^). The mean daily food consumption of adults was 1.5 times higher than that of children (1900 and 1228 g/d/person, respectively). Thus, adults exhibited higher ^134^Cs+^137^Cs intake than children (p < 0.001).

**Table 2:** Dietary exposure to ^134^Cs and ^137^Cs in Japan estimated using the duplicate portion study

**^134^Cs + ^137^Cs**	**Region^1^**	**Age group^2^**	**FY^3^**	**n**	**Range**	**Mean**	**SD**
Daily intake (Bq/d/person)	Tohoku–Kanto	Adults	2012	36	0.024–1.1	0.25	0.27
			2013	36	0.052–2.2	0.31	0.40
			2014	36	0.028–1.0	0.20	0.23

		Children	2012	31	0.024–1.9	0.24	0.39
			2013	29	0.037–19	0.80	3.5
			2014	29	0.021–0.48	0.14	0.11

	Others	Adults	2012	9	0.023–0.34	0.12	0.11
			2013	9	0.022–0.26	0.12	0.080
			2014	9	0.028–0.25	0.083	0.068

		Children	2012	6	0.014–0.055	0.033	0.015
			2013	6	0.02–0.063	0.038	0.016
			2014	6	0.026–0.072	0.042	0.017

Committed effective dose from one year’s intake (µSv)	Tohoku–Kanto	Adults	2012	36	0.14–6.0	1.4	1.5
			2013	36	0.28–12	1.7	2.2
			2014	36	0.17–5.3	1.1	1.2

		Children	2012	31	0.1–7.5	0.96	1.5
			2013	29	0.15–74	3.1	14
			2014	29	0.085–1.8	0.54	0.41

	Others	Adults	2012	9	0.13–1.9	0.62	0.57
			2013	9	0.14–1.4	0.61	0.44
			2014	9	0.16–1.3	0.45	0.36

		Children	2012	6	0.056–0.21	0.13	0.058
			2013	6	0.08–0.18	0.12	0.040
			2014	6	0.11–0.28	0.17	0.059

The values listed in this table were calculated assuming values of half the limit of detection for non-detects. Committed effective doses were estimated assuming a one-year intake of the samples.

^1^The Tohoku–Kanto region includes Iwate, Miyagi, Fukushima, Ibaraki, Saitama, Tokyo, and Kanagawa, and the others include Hokkaido, Osaka, and Kochi.

^2^Adults and children were persons aged 20 years and over, and aged three–six years, respectively.

^3^Fiscal year. The sampling periods for FY 2012, FY 2013, and FY 2014 were March 2013, September–November 2013, and December 2014–March 2015, respectively.

The top five CEDs due to ^134^Cs+^137^Cs were 74, 12, 7.5, 6.8, and 6.0 µSv. Although the highest individual CED (children in Date City, FY 2013) was six times higher than the second-highest, the value was 14 times lower than the annual maximum permissible level of 1 mSv. The ^134^Cs+^137^Cs CEDs in the Tohoku–Kanto region significantly exceeded those in the other regions (p < 3 × 10^−10^). Adults exhibited higher ^134^Cs+^137^Cs CEDs than children (p < 5 × 10^−9^) because the adult ^134^Cs and ^137^Cs dose coefficients are higher than those for 5-year-old children [15] and the adult intake of ^134^Cs+^137^Cs was higher, as previously explained. Significant correlations were not observed between the ^134^Cs+^137^Cs intakes and CEDs and the fiscal years (p < 0.5, p < 0.3, respectively). However, analysis of the annual change was difficult because some of the participant households changed.

Selection bias constitutes one limitation of this study; that is, participants may avoid foods produced near the FDNPP and underestimate the radionuclide exposure levels. However, the questionnaire data revealed that 29% and 43% of the respondents in Fukushima Prefecture were not concerned about radioactive materials when buying foods and were consuming homegrown vegetables, respectively. Thus, the findings of this study were not substantially underestimated. Another limitation is that the study sample size was not well-considered, as the study was designed to evaluate radionuclide dietary intake. Regardless, the results demonstrate that the Tohoku–Kanto region and adults exhibited significantly higher ^134^Cs+^137^Cs CEDs than other regions and children, respectively.

### ^90^Sr

^90^Sr was detected in 34 of the 78 DP samples. The activity concentrations, dietary intakes, and CEDs of ^90^Sr are summarized in Table 3. The ^90^Sr maximum, median, and mean activity concentrations were 0.015–0.040, 0.010–0.019, and 0.012–0.017 Bq/kg, respectively. Adults had higher dietary intake of ^90^Sr than children (p < 3 × 10^−8^) owing to their higher quantity of daily food consumption. The CEDs due to ^90^Sr in adults and children did not differ significantly (95% CI, −0.011–infinity; p < 0.1), and the ^90^Sr dose coefficient for children exceeded that for adults (4.7 × 10^−8^ and 2.8 × 10^−8^ Sv/Bq, respectively). As the DP sample numbers in the other regions was too small, significant differences were not apparent in the ^90^Sr intakes and CEDs between regions (95% CI, −0.0049–0.0079; p < 0.6, 95% CI, −0.035–0.089; p < 0.4, respectively). The highest CED due to ^90^Sr was 0.66 µSv (Table 3), which was approximately 110 times lower than that due to ^134^Cs+^137^Cs and 1,500 times lower than the maximum permissible level.

**Table 3:** Dietary exposure to ^90^Sr in Japan estimated using the duplicate portion study

**^90^Sr**	**Region^1^**	**Age group^2^**	**FY^3^**	**n**	**Range**	**Mean**	**SD**	**Detection rate (%)**
Activity concentration (Bq/kg)	Tohoku–Kanto	Adults	2012	11	0.010–0.022	0.013	0.0041	36
			2013	14	0.010–0.028	0.015	0.0061	43
			2014	14	0.010–0.024	0.017	0.0050	79

		Children	2012	9	0.010–0.015	0.012	0.0026	11
			2013	11	0.010–0.040	0.016	0.010	18
			2014	10	0.010–0.023	0.016	0.0053	60

	Others	Adults	2012	3	0.010–0.022	0.016	0.0060	67
			2013	3	0.010–0.023	0.014	0.0075	33
			2014	3	0.010–0.024	0.015	0.0081	33

Daily intake (Bq/d/person)	Tohoku–Kanto	Adults	2012	11	0.014–0.046	0.024	0.011	-
			2013	14	0.014–0.062	0.030	0.017	-
			2014	14	0.011–0.050	0.032	0.012	-

		Children	2012	9	0.0080–0.021	0.014	0.0046	-
			2013	11	0.012–0.026	0.017	0.0043	-
			2014	10	0.0070–0.030	0.018	0.0071	-

	Others	Adults	2012	3	0.021–0.053	0.041	0.018	-
			2013	3	0.022–0.044	0.030	0.012	-
			2014	3	0.023–0.065	0.041	0.022	-

Committed effective dose from one year’s intake (µSv)	Tohoku–Kanto	Adults	2012	11	0.14–0.47	0.24	0.12	-
			2013	14	0.15–0.64	0.31	0.17	-
			2014	14	0.11–0.51	0.32	0.13	-

		Children	2012	9	0.14–0.35	0.24	0.08	-
			2013	11	0.20–0.44	0.28	0.07	-
			2014	10	0.12–0.51	0.31	0.12	-

	Others	Adults	2012	3	0.21–0.54	0.42	0.18	-
			2013	3	0.22–0.45	0.31	0.12	-
			2014	3	0.23–0.66	0.42	0.22	-

The values listed in this table were calculated assuming values of half the limit of detection for non-detects. Committed effective doses were estimated assuming a one-year intake of the samples.

^1^The Tohoku–Kanto region includes Iwate, Miyagi, Fukushima (Soma City and Fukushima City), and Tokyo and the others include Kochi. In FY 2014, samples were collected from Minamisoma City instead of Soma City.

^2^Adults and children were persons aged 20 years and over, and aged three to six years, respectively.

^3^Fiscal year. The sampling periods for FY 2012, FY 2013, and FY 2014 were March 2013, September–November 2013 and December 2014–March 2015, respectively.

### ^239^Pu and ^240^Pu

The total atmospheric release of ^239^Pu and ^240^Pu during the FDNPP accident was seven orders of magnitude lower than that of ^134^Cs and ^137^Cs (Supplementary Table S1). Here, ^239+240^Pu was not detected in any DP samples. Supplementary Table S4 outlines the ^239+240^Pu results, which were calculated using the values of half the LODs for non-detects. The maximum activity concentration and dietary intake were 0.00044 and 0.00071 Bq/d/person, respectively. The maximum CED of ^239+240^Pu was 0.065 Sv: 10 and 1,100 times lower than those of ^90^Sr and ^134^Cs+^137^Cs, respectively.

### DP study conducted before accident

**Fig. 2:**
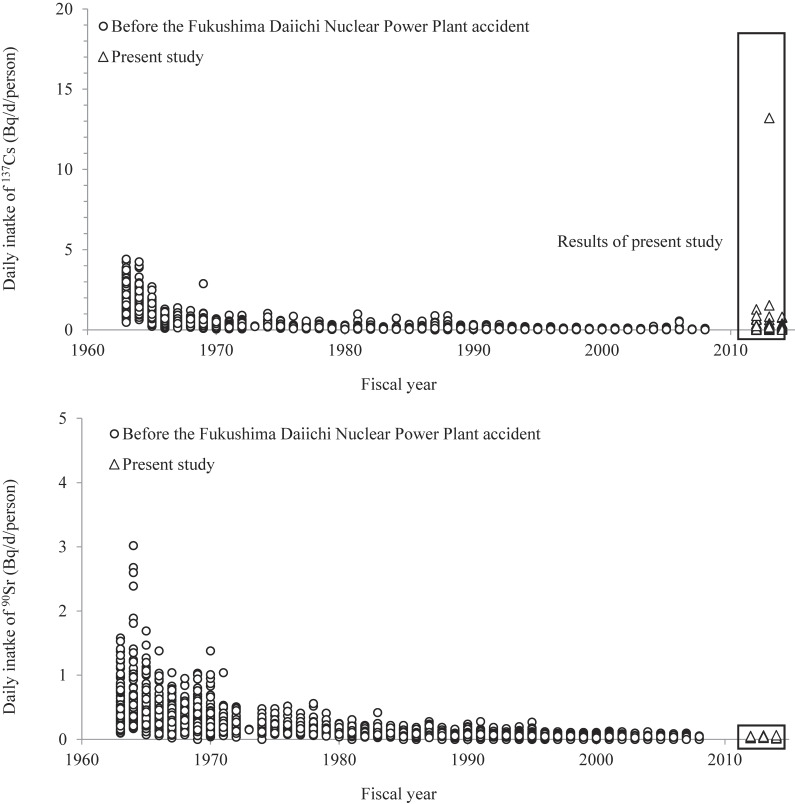
Dietary intakes of ^137^Cs and ^90^Sr in Japan estimated using duplicate portion studies Open circles represent data on radionuclide intakes during 1963–2008 obtained from the following source: Environmental Radiation Database. https://www.kankyo-hoshano.go.jp/en/data-en/database-en/ Open triangles represent data from the present study.

Figure 2 shows the ^137^Cs intakes obtained in this study and from the “Environmental Radiation Database,” which includes FY 1963–2008 intake data for Japan obtained using the DP approach [19, 20]. In the early 1960s, the intake was relatively high because many nuclear weapon tests were conducted and radionuclides were released into the atmosphere. Before the FDNPP accident, the highest intake value was 4.4 Bq/d/person in 1963; this reduced to 0.0071–0.56 Bq/d/person during 1999–2008 (the decade before the accident) [20]. Although ^137^Cs intake increased sharply owing to the FDNPP accident, the results from the present study are lower than 4.4 Bq/d/person, with just one exception. In FY 2014, the intake decreased to below 0.5 Bq/d/person in 78 out of 80 DP samples, which was in the range observed during 1999–2008. The ^90^Sr intakes for FY 1963–2014 are shown in Fig. 2. Similar to ^137^Cs, the ^90^Sr intakes were higher in the early 1960s because of nuclear testing. The highest ^90^Sr intake was 3.0 Bq/d/person in 1964, higher than that found in this study (0.065 Bq/d/person) [20]. Because of the FDNPP accident, environmental samples collected from Fukushima Prefecture before and after the accident display significant differences in ^90^Sr concentrations [21]. However, the ^90^Sr intake after the accident did not increase considerably and was similar to that observed for 1999–2008 (0.02–0.13 Bq/d/person); the total ^90^Sr release into the atmosphere by the FDNPP accident was approximately 100 times lower than that of ^134^Cs and ^137^Cs (Supplementary Table S1). Thus, no effect of the FDNPP accident on ^90^Sr intake was observed in this study.

### DP studies conducted after accident

Many studies evaluated radionuclide dietary exposure after the FDNPP accident [22–36]. Supplementary Table S5 lists the ^134^Cs+^137^Cs CEDs of those studies. The exposure levels immediately after the FDNPP accident were not estimated as the present study was conducted approximately two years later. The CEDs from this study were compared with those from other DP studies conducted prior to the accident (Supplementary Table S5). Koizumi et al. [22] and Harada et al. [23] observed maximum CEDs in the Fukushima Prefecture of 83.1 and 99 µSv in July and December 2011, respectively. The mean CEDs were 3.0 and 26 µSv in July and December 2011, respectively. The differences were presumably due to seasonal variations or the employed survey methods [22, 23]. In the present study, seasonal variations in CEDs could not be assessed because sampling was performed in one season only. Hirokawa et al. [24] collected DP samples from 18 prefectures during November 2011–February 2015, and reported a maximum CED of ^134^Cs+^137^Cs 140 µSv, which was comparable to those observed by Koizumi et al. [22] and Harada et al. [23].

The Fukushima Prefecture has been conducting similar studies since FY 2012. The maximum ^134^Cs+^137^Cs activity concentration in the DP samples for FY 2012 was 150 Bq/kg, exceeding the standard limit for general foods (100 Bq/kg). These high ^134^Cs+^137^Cs concentrations were attributed to wild mushrooms and homegrown ingredients [25]. The CED from the annual consumption of this DP sample was 2.1 mSv, exceeding the annual maximum permissible level of 1 mSv. When this sample was excluded, the maximum CED due to ^134^Cs+^137^Cs in FY 2012 was estimated at 120 µSv [25], comparable to the results described above. The FY 2011 and 2012 results exhibited higher maximum CEDs due to ^134^Cs+^137^Cs than those of the present study; however, the CEDs were sufficiently lower than the maximum permissible level, with one exception (Supplementary Table S5).

Ichihashi et al. [26] and Takuma et al. [27] performed similar studies in the Hokkaido and Kochi Prefectures, respectively, and reported maximum CEDs due to ^134^Cs+^137^Cs of 22 and 2.0 µSv, respectively. Their and the present results indicate that the CEDs due to ^134^Cs+^137^Cs in the other regions are lower than those in the Tohoku–Kanto region (Supplementary Table S5).

A DP study of ^90^Sr and ^239+240^Pu was conducted by the MHLW in Spring 2012. The results indicated that the ^90^Sr maximum activity concentration and dietary intake were 0.24 Bq/kg and 0.0060 Bq/d/person, respectively [28], both of which are comparable to our results. A study in the Fukushima Prefecture conducted since FY 2012 revealed a maximum ^90^Sr intake of 0.12 Bq/d/person (February–April 2013) [25], exceeding that obtained in the present study and in the range of the ^90^Sr intake for 1999–2008. ^239+240^Pu was not detected in any of the DP samples of either study. Other DP study of ^90^Sr was conducted by Harada et al. [29]. They indicated that DP samples in Kawauchi village, the Fukushima Prefecture did not show detectable amount of ^90^Sr and ^89^Sr.

### TDS conducted after accident

One disadvantage of DP studies is that, unlike TDSs, they cannot identify the chief dietary sources of contamination [8, 9]. Our previous study, which was performed using the market basket method in FY 2011, indicated that the main sources of CEDs due to ^134^Cs+^137^Cs were milk and dairy products, fish and shellfish, rice and rice products, and fruits [7]. Similar results were obtained by Tsutsumi et al. [30].

In the DP studies, some samples exhibited high ^134^Cs+^137^Cs activity concentrations because of their homegrown ingredients. The TDS samples did not include homegrown ingredients because they were collected from supermarkets. Therefore, the TDS showed lower CED values than the DP studies. The maximum CEDs due to ^134^Cs+^137^Cs obtained in the DP studies and TDS were 140 and 19 µSv in FY 2011, 2100 and 7.1 µSv in FY 2012, 74 and 2.7 µSv in FY 2013, and 10 and 2.2 µSv in FY 2014, respectively (Supplementary Table S5).

The MHLW and Tokyo Metropolitan Government have conducted TDSs of ^134^Cs+^137^Cs since FY 2011 [30–35]. The most recent MHLW results (September–October 2022) indicate that the ^134^Cs+^137^Cs CED has decreased to 1.1 µSv in Fukushima Prefecture [35], which is 17 times lower than that in FY 2011. The MHLW also performed the TDS of ^90^Sr from FY 2013 and showed that the highest estimated CED due to ^90^Sr was 0.76 µSv [36], which was comparable to the results of DP studies and the TDS conducted before the FDNPP accident [12].

## Conclusion

In the present study, the CEDs due to ^134^Cs+^137^Cs were lower in children than in adults. Regardless, the highest CED due to ^134^Cs+^137^Cs, i.e., 74 µSv, was observed in the DP of a child. However, this value was approximately 14 times lower than the maximum permissible level of 1 mSv/y. The ^90^Sr intake was at the same level as that before the FDNPP accident, and ^239+240^Pu was not detected in any of the analyzed samples. Thus, the dietary exposure levels of ^134^Cs+^137^Cs, ^90^Sr, and ^239+240^Pu observed in the present study were sufficiently lower than the regulatory levels and may not pose a health risk. DP study has advantage over TDS that it can reflect individual preference for food. In this study, we could indicate that the highest concentration of the DP sample was due to homegrown foods. In addition, there are limited reports on dietary intake of ^90^Sr and Pu, especially on Pu intake. Thus, our findings are valuable and helpful for radiological safety of foods.

## Abbreviations

CED: committed effective dose
DP: duplicate portion
FDNPP: Fukushima Daiichi Nuclear Power Plant
FY: fiscal year
LOD: limit of detection
MHLW: Ministry of Health, Labour and Welfare
NRA: Nuclear Regulation Authority
TDS: total diet study

## Supplementary information

**Additional file 1:** **Supplementary Table S1** Total amounts of radionuclides released into the atmosphere from the Fukushima Daiichi Nuclear Power Plant. **Supplementary Table S2** Dietary questionnaire (for adults). **Supplementary Table S3** Dietary questionnaire (for participants with children). **Supplementary Table S4** Dietary exposure to ^239+240^Pu in Japan estimated by the duplicate portion study. **Supplementary Table S5** Estimated committed effective doses of ^134+137^Cs in Japan after the Fukushima Daiichi Nuclear Power Plant accident.

## Declarations

### Availability of data and material

Please contact the corresponding author for data requests.
