# Supplementary material for: Dietary exposure levels to 134Cs, 137Cs, 90Sr, and 239+240Pu in Japan after the Fukushima Daiichi Nuclear Power Plant accident: a duplicate portion study for fiscal years 2012–2014
Source: Environ Health Prev Med. 2025 Jun 25;30:48. doi: 10.1265/ehpm.25-00072 (PMC12206664; doi:10.1265/ehpm.25-00072)
Supplement: Supplementary file 1 — Additional file 1: Supplementary Table S1 Total amounts of radionuclides released into the atmosphere from the Fukushima Daiichi Nuclear Power Plant. Supplementary Table S2 Dietary questionnaire (for adults). Supplementary Table S3 Dietary questionnaire (for participants with children). Supplementary Table S4 Dietary exposure to 239+240Pu in Japan estimated by the duplicate portion study. Supplementary Table S5 Estimated committed effective doses of 134+137Cs in Japan after the Fukushima Daiichi Nuclear Power Plant accident. [file ehpm-30-048-s001.docx]

**Supplementary Materials**

**For**

**Dietary exposure levels to ^134^Cs, ^137^Cs, ^90^Sr, and ^239+240^Pu in Japan after the Fukushima Daiichi Nuclear Power Plant accident: A duplicate portion study for fiscal years 2012–2014**

Hiroshi Terada^1, *^, Ikuyo Iijima^2^, Sadaaki Miyake^3^, Tomoko Ota^4^, Ichiro Yamaguchi^1^, Hiroko Kodama^5^, Naoki Kunugita^1, 6^ and Hideo Sugiyama^5^

^1^ National Institute of Public Health, 2-3-6 Minami, Wako-shi, Saitama 351-0197, Japan

^2^ Kanagawa Prefectural Institute of Public Health, 1-3-1 Shimomachiya, Chigasaki-shi, Kanagawa 253-0087, Japan

^3^ Saitama Prefectural Institute of Public Health, 410-1 Ewai, Yoshimi-machi, Hiki-gun, Saitama 355-0133, Japan

^4^ Japan Chemical Analysis Center, 295-3, Sanno-cho, Inage-ku, Chiba-shi, Chiba 263-0002, Japan

^5^ Teikyo Heisei University, 2-51-4 Higashiikebukuro, Toshima-ku, Tokyo, 170-8445, Japan

^6^ Current address: School of Health Sciences, University of Occupational and Environmental Health, 1-1 Iseigaoka, Yahatanishi, Kitakyushu-shi, Fukuoka 807-8555, Japan

**Corresponding author:** Hiroshi Terada; e-mail: terada.h.aa@niph.go.jp

**Supplementary Table S1** Total amounts of radionuclides released into the atmosphere from the Fukushima Daiichi Nuclear Power Plant

| **Radionuclide^1^** | **Total release (PBq) ^2^** | **Half-life^3^** |
| --- | --- | --- |
| ^133^Xe | 11000 | 5.2475 d |
| ^131^I | 160 | 8.0252 d |
| ^132^Te | 88 | 3.204 d |
| ^133^I | 42 | 20.83 h |
| ^134^Cs | 18 | 2.0652 y |
| ^137^Cs | 15 | 30.08 y |
| ^127^Sb | 6.4 | 3.85 d |
| ^131m^Te | 5.0 | 33.25 h |
| ^129m^Te | 3.3 | 33.6 d |
| ^140^Ba | 3.2 | 12.751 d |
| ^135^I | 2.3 | 6.58 h |
| ^89^Sr | 2.0 | 50.563 d |
| ^127m^Te | 1.1 | 106.1 d |
| ^90^Sr | 0.14 | 28.91 y |
| ^241^Pu | 0.0012 | 14.290 y |
| ^238^Pu | 0.000019 | 87.7 y |
| ^239^Pu | 0.0000032 | 24110 y |
| ^240^Pu | 0.0000032 | 6561 y |
| ^106^Ru | 0.0000021 | 371.8 d |

^1^ This table lists radionuclides that have half-lives exceeding one year or have been released in quantities greater than 1 PBq.

^2^ Obtained from the following reference:

Nuclear and Industrial Safety Agency. Regarding the evaluation of the conditions on reactor cores of Unit 1,2 and 3 related to the accident at Fukushima Dai-ichi Nuclear Power Station, Tokyo Electric Power Co. Inc. (revised at 20 October 2011). http://warp.da.ndl.go.jp/info:ndljp/pid/8422823/www.meti.go.jp/press/2011/06/20110606008/20110606008-2.pdf. Accessed 7 March 2025. [in Japanese]

^3^ Obtained from the following reference:

National Nuclear Data Center. Decay Radiation Database, version 1/13/2023. <https://www.nndc.bnl.gov/nudat3/indx_dec.jsp>. Accessed 7 March 2025.

**Supplementary Table S2** Dietary questionnaire (for adults)

1. Menu table

Please fill in the table below with the menu for each meal.

| Sampling date | Breakfast | Lunch | Dinner | Snacks (if any) |
| --- | --- | --- | --- | --- |
| Day 1  （Month/day） |  |  |  |  |
| Day 2  （Month/day） |  |  |  |  |

2. Food items

Please fill in a circle on all of the following ingredients used in the meals.

| Ingredient | Day 1 | Day 2 |
| --- | --- | --- |
| Grains (rice, wheat, buckwheat, corn, miscellaneous grains) and their products (rice flour, rice noodle, bread, pasta, udon, dumpling skin, etc.) |  |  |
| Potatoes (potatoes, sweet potatoes, etc.) and their products (potato starch, glass noodles, etc.) |  |  |
| Beans and their products (soybeans, soybean flour, tofu, soy milk, red beans, sweet bean paste, etc.). Miso is not included. |  |  |
| Green and yellow vegetables (tomatoes, spinach, etc.) including vegetable juice |  |  |
| Vegetables other than green and yellow vegetables (cucumber, lettuce, cabbage, etc.) |  |  |
| Fruits (including jams, juices, dried fruits, etc.) |  |  |
| Mushrooms |  |  |
| Seafood (fish, shellfish, squid, shrimp, octopus, etc.) and their products (chikuwa, fish cake, etc.) |  |  |
| Seaweeds (wakame, kelp, hijiki, etc.) |  |  |
| Wild game meat (wild boar, deer, bear, etc.) |  |  |
| Dairy products (milk, cheese, yogurt, ice cream, etc.) |  |  |
| Beverages (green tea, barley tea, coffee, cocoa, carbonated drinks, etc.) |  |  |

3. Please answer the following questions.

Q1. Are you currently concerned about radioactive materials when buying food?

1. Very concerned

2. Somewhat concerned

3. Neutral

4. Not too concerned

5. Not at all concerned

Q2. For those who answered 1 or 2 in Q1, please fill in the blank with any foods produced in Fukushima and neighboring prefectures that you currently avoid purchasing in relation to the nuclear power plant accident.

Q3: Do you currently consume homegrown vegetables?

1. Yes (item: ) 2. No

*If homegrown vegetables were used in your meals, please write "home" in place of circle in the table of 2. Food items.

Q4. Please indicate your gender.

1. female 2. male

Q5. How old are you?

1. 20s 2. 30s 3. 40s 4. 50s 5. 60 or older

This is the end of the questionnaire. Thank you for your cooperation.

**Supplementary Table S3** Dietary questionnaire (for participants with children)

1. Menu table

Please fill in the table below with the menu for each meal.

| Sampling date | Breakfast | Lunch | Dinner | Snacks (if any) |
| --- | --- | --- | --- | --- |
| Day 1  （Month/day） |  |  |  |  |
| Day 2  （Month/day） |  |  |  |  |

2. Food items

Please fill in a circle on all of the following ingredients used in the meals.

| Ingredient | Day 1 | Day 2 |
| --- | --- | --- |
| Grains (rice, wheat, buckwheat, corn, miscellaneous grains) and their products (rice flour, rice noodle, bread, pasta, udon, dumpling skin, etc.) |  |  |
| Potatoes (potatoes, sweet potatoes, etc.) and their products (potato starch, glass noodles, etc.) |  |  |
| Beans and their products (soybeans, soybean flour, tofu, soy milk, red beans, sweet bean paste, etc.). Miso is not included. |  |  |
| Green and yellow vegetables (tomatoes, spinach, etc.) including vegetable juice |  |  |
| Vegetables other than green and yellow vegetables (cucumber, lettuce, cabbage, etc.) |  |  |
| Fruits (including jams, juices, dried fruits, etc.) |  |  |
| Mushrooms |  |  |
| Seafood (fish, shellfish, squid, shrimp, octopus, etc.) and their products (chikuwa, fish cake, etc.) |  |  |
| Seaweeds (wakame, kelp, hijiki, etc.) |  |  |
| Wild game meat (wild boar, deer, bear, etc.) |  |  |
| Dairy products (milk, cheese, yogurt, ice cream, etc.) |  |  |
| Beverages (green tea, barley tea, coffee, cocoa, carbonated drinks, etc.) |  |  |

3. Please answer the following questions.

Q1. Are you currently concerned about radioactive materials when buying food?

1. Very concerned

2. Somewhat concerned

3. Neutral

4. Not too concerned

5. Not at all concerned

Q2. For those who answered 1 or 2 in Q1, please fill in the blank with any foods produced in Fukushima and neighboring prefectures that you currently avoid purchasing in relation to the nuclear power plant accident.

Q3: Do you currently cousume homegrown vegetables?

1. Yes (item: ) 2. No

*If homegrown vegetables were used in your meals, please write "home" in place of circle in the table of 2. Food items.

Q4. Please indicate your child's gender.

1. female 2. male

Q5. How old is your child?

1. three years old 2. four years old 3. five years old 4. six years old

Q5. what is your child's weight?

1. Below 14 kg 2. 14–16 kg 3. 16–18 kg 4. 18–20 kg 5. Over 20 kg

This is the end of the questionnaire. Thank you for your cooperation.

**Supplementary Table S4** Dietary exposure to ^239+240^Pu in Japan estimated by the duplicate portion study

| **^239+240^Pu** | **Region ^1^** | **Age group ^2^** | **FY ^3^** | **n** | **Max.** | **Min.** | **Median** | **Mean** | **SD** |
| --- | --- | --- | --- | --- | --- | --- | --- | --- | --- |
| Activity concentration (Bq/kg) | Tohoku–Kanto | Adults | 2013 | 15 | 0.00029 | 0.00020 | 0.00022 | 0.00022 | 0.000025 |
|  |  |  | 2014 | 15 | 0.00026 | 0.00009 | 0.00022 | 0.00022 | 0.000037 |
|  |  | Children | 2013 | 11 | 0.00025 | 0.00013 | 0.00021 | 0.00019 | 0.000045 |
|  |  |  | 2014 | 12 | 0.00044 | 0.00012 | 0.00022 | 0.00025 | 0.00010 |
|  | Others | Adults | 2013 | 3 | 0.00022 | 0.00020 | 0.00021 | 0.00021 | 0.000008 |
|  |  |  | 2014 | 3 | 0.00023 | 0.00021 | 0.00023 | 0.00022 | 0.000012 |
| Daily intake  (Bq/d/person) | Tohoku–Kanto | Adults | 2013 | 15 | 0.00064 | 0.00022 | 0.00046 | 0.00044 | 0.00012 |
|  |  |  | 2014 | 15 | 0.00062 | 0.00021 | 0.00043 | 0.00041 | 0.00011 |
|  |  | Children | 2013 | 11 | 0.00034 | 0.00006 | 0.00023 | 0.00023 | 0.000070 |
|  |  |  | 2014 | 12 | 0.00040 | 0.00016 | 0.00025 | 0.00027 | 0.000070 |
|  | Others | Adults | 2013 | 3 | 0.00055 | 0.00039 | 0.00045 | 0.00046 | 0.000078 |
|  |  |  | 2014 | 3 | 0.00071 | 0.00051 | 0.00062 | 0.00062 | 0.00010 |
| Committed effective dose from one year’s intake (μSv) | Tohoku–Kanto | Adults | 2013 | 15 | 0.058 | 0.020 | 0.042 | 0.040 | 0.011 |
|  |  |  | 2014 | 15 | 0.057 | 0.019 | 0.039 | 0.037 | 0.010 |
|  |  | Children | 2013 | 11 | 0.041 | 0.007 | 0.027 | 0.027 | 0.0085 |
|  |  |  | 2014 | 12 | 0.048 | 0.020 | 0.03075 | 0.032 | 0.0084 |
|  | Others | Adults | 2013 | 3 | 0.050 | 0.036 | 0.041 | 0.042 | 0.0071 |
|  |  |  | 2014 | 3 | 0.065 | 0.047 | 0.057 | 0.056 | 0.0093 |

None of the DP samples contained detectable levels of ^239+240^Pu. The values listed in this table were calculated assuming values of half the limit of detection for non-detects. Committed effective doses were estimated assuming a one-year intake of the samples.

^1^ The Tohoku–Kanto region includes Iwate, Miyagi, Fukushima (Souma City and Fukushima City), and Tokyo and the Others region includes Kochi. In FY 2014, samples were collected from Minamisouma City instead of Souma City.

^2^ Adults and children were persons aged 20 years and over, and aged three to six years, respectively.

^3^ Fiscal year. The sampling periods for FY 2012, FY 2013, and FY 2014 were March 2013, September–November 2013 and December 2014–March 2015, respectively.

**Supplementary Table S5** Estimated committed effective doses of ^134+137^Cs in Japan after the Fukushima Daiichi Nuclear Power Plant accident.

| **Type of study** | **Sampling period** | | **Region** | **Max. dose (μSv)** | **Reference** |
| --- | --- | --- | --- | --- | --- |
| Duplicate portion | FY^1^ 2011 | July | Tohoku (Fukushima) | 83.1 | [22] |
|  |  | July | Others (Kyoto) | 5.3 | [22] |
|  |  | July | Others (Hokkaido) | 6 | [26] |
|  |  | November | Others (Hokkaido) | 7.3 | [26] |
|  |  | November-March | Tohoku, Kanto, Others^2^ | 140 | [24] |
|  |  | December | Tohoku, Kanto^3^ | 99 | [23] |
|  |  | December | Others^4^ | 3.6 | [23] |
|  |  | February | Others (Hokkaido) | 22 | [26] |
|  |  | March–May | Tohoku, Kanto^5^ | 5.4 | [30] |
|  |  | March–May | Others (Hokkaido, Osaka, Kochi) | 1.4 | [30] |
|  | FY 2012 | June | Others (Kochi) | 2.0 | [27] |
|  |  | June | Tohoku (Fukushima) | 14 | [25] |
|  |  | July | Others (Hokkaido) | 1.9 | [26] |
|  |  | September | Others (Kochi) | 0.33 | [27] |
|  |  | September–November | Tohoku (Fukushima) | 2100 | [25] |
|  |  | October–February | Tohoku (Fukushima) | 53 | [25] |
|  |  | December–February | Tohoku (Fukushima) | 120 | [25] |
|  |  | February–April | Tohoku (Fukushima) | 16 | [25] |
|  |  | March | Others (Kochi) | 0.11 | [27] |
|  |  | March | Tohoku, Kanto^6^ | 7.5 | Present study |
|  |  | March | Others (Hokkaido, Osaka, Kochi) | 1.9 | Present study |
|  |  |  | Tohoku, Kanto, Others^2^ | 53 | [24] |
|  | FY 2013 | September November | Tohoku, Kanto^6^ | 74 | Present study |
|  |  | September–November | Others (Hokkaido, Osaka, Kochi) | 1.4 | Present study |
|  |  |  | Tohoku (Fukushima) | 28 | [25] |
|  |  |  | Tohoku, Kanto, Others^2^ | 32 | [24] |
|  | FY 2014 | December–March | Tohoku, Kanto^6^ | 5.3 | Present study |
|  |  | December–March | Others (Hokkaido, Osaka, Kochi) | 1.3 | Present study |
|  |  |  | Tohoku (Fukushima) | 10 | [25] |
|  | FY 2015–2021 |  | Tohoku (Fukushima) | 23 | [25] |
| Total diet | FY 2011 | August | Others (Nagoya) | 1.5 | [37] |
|  |  | September–November | Tohoku, Kanto (Miyagi, Fukushima, Tokyo) | 19 | [30] |
|  |  | October–November | Tohoku, Kanto (Miyagi, Fukushima, Tokyo) | 17 | [10] |
|  |  | March | Tohoku, Kanto, Others^7^ | 9.4 | [31] |
|  |  |  | Kanto (Tokyo) | 3.3 | [34] |
|  | FY 2012 | September | Tohoku, Kanto, Others^7^ | 3.8 | [31] |
|  |  | March | Tohoku, Kanto, Others^7^ | 7.1 | [31] |
|  |  |  | Kanto (Tokyo) | 1.3 | [34] |
|  | FY 2013 | September | Tohoku, Kanto, Others^7^ | 2.7 | [32] |
|  |  | March | Tohoku, Kanto, Others^7^ | 1.9 | [31] |
|  |  |  | Kanto (Tokyo) | 0.4 | [34] |
|  | FY 2014 | September | Tohoku, Kanto, Others^7^ | 2.2 | [32] |
|  |  | March | Tohoku, Kanto, Others^7^ | 2.0 | [32] |
|  |  |  | Kanto (Tokyo) | 0.63 | [34] |
|  | FY 2015–2022 | | Tohoku, Kanto, Others^7^ | 1.5 | [32, 34] |
|  | FY 2015–2021 | | Kanto (Tokyo) | 0.72 | [34] |

Committed effective doses were estimated assuming a one-year intake of the samples.

^1^ Fiscal year

^2^ Iwate, Miyagi, Fukushima, Gunma, Tochigi, Ibaraki, Saitama, Tokyo, Chiba, Kanagawa, Yamanashi, Nagano, Niigata, Shizuoka, Aichi, Gifu, Mie, and Fukuoka

^3^ Fukushima, Ibaraki, Tochigi, Gunma, Saitama, Chiba, Tokyo, Kanagawa, and Nagano

^4^ Gifu, Aichi, Mie, Kyoto, Osaka, and Fukuoka

^5^ Iwate, Fukushima, Ibaraki, Tochigi, Saitama, and Niigata.

^6^ Iwate, Miyagi, Fukushima, Ibaraki, Saitama, Tokyo, and Kanagawa

^7^ Hokkaido, Iwate, Miyagi, Fukushima, Ibaraki, Tochigi, Saitama, Tokyo, Kanagawa, Niigata, Osaka, Kochi, and Nagasaki

Supplementary references

1. Miyazaki H, Kato H, Kato Y, Tsuchiyama T, Terada H. Estimation of the intake of radioactive cesium based on analysis of total diet samples in Nagoya. J Food Hyg Soc Jpn. 2013;54:151–5. [in Japanese]
